# Continuous and discrete-time survival prediction with neural networks

**DOI:** 10.1007/s10985-021-09532-6

**Published:** 2021-10-07

**Authors:** Håvard Kvamme, Ørnulf Borgan

**Affiliations:** Department of Mathematics, University of Oslo, P.O. Box 1053 Blindern, 0316 Oslo, Norway

**Keywords:** Time-to-event prediction, Neural networks, Interpolation, Discretization

## Abstract

Due to rapid developments in machine learning, and in particular neural networks, a number of new methods for time-to-event predictions have been developed in the last few years. As neural networks are parametric models, it is more straightforward to integrate parametric survival models in the neural network framework than the popular semi-parametric Cox model. In particular, discrete-time survival models, which are fully parametric, are interesting candidates to extend with neural networks. The likelihood for discrete-time survival data may be parameterized by the probability mass function (PMF) or by the discrete hazard rate, and both of these formulations have been used to develop neural network-based methods for time-to-event predictions. In this paper, we review and compare these approaches. More importantly, we show how the discrete-time methods may be adopted as approximations for continuous-time data. To this end, we introduce two discretization schemes, corresponding to equidistant times or equidistant marginal survival probabilities, and two ways of interpolating the discrete-time predictions, corresponding to piecewise constant density functions or piecewise constant hazard rates. Through simulations and study of real-world data, the methods based on the hazard rate parametrization are found to perform slightly better than the methods that use the PMF parametrization. Inspired by these investigations, we also propose a continuous-time method by assuming that the continuous-time hazard rate is piecewise constant. The method, named PC-Hazard, is found to be highly competitive with the aforementioned methods in addition to other methods for survival prediction found in the literature.

## Introduction

Survival analysis considers the problem of modeling the distribution of the time to an event. A plethora of statistical methods for analyzing time-to-event data, and especially right-censored survival data, have been developed over the last fifty years or so. Most of these methods, like Cox regression, assume continuous-time models, but methods based on discrete-time models are sometimes used as well. Statistical methods for continuous-time survival data are discussed in a number of textbooks, such as Klein and Moeschberger (2003). The literature on discrete-time models and methods is more limited, but the recent book by Tutz and Schmid (2016) provides a nice presentation.

An important part of survival analysis is the topic of *time-to-event prediction*, also denoted *survival prediction*. This generally concerns predicting when an event will occur for new individuals (not part of our training set), where each individual is defined by a vector of covariates. Time-to-event prediction is most commonly approached by predicting the survival function for each individual, meaning we provide an estimate of the event time distribution conditioned on each individual’s covariates. In standard survival analysis, Cox regression is often used for this purpose (Klein and Moeschberger 2003, Chapter 8.6).

As a result of the rapid development in machine learning, and in particular neural networks, a number of new methods for time-to-event predictions have been developed in the last few years. This development has benefited from the excellent frameworks for neural network development, such as TensorFlow, PyTorch, Theano, Keras, and CNTK, which have simplified the application of neural networks to existing likelihood-based methodologies. Thus, novel methods for time-to-event predictions have been developed based on Cox’s partial likelihood (e.g., Katzman et al. 2018; Luck et al. 2017; Yousefi et al. 2017; Kvamme et al. 2019) and the likelihood for discrete-time survival data (e.g., Lee et al. 2018; Fotso 2018; Gensheimer and Narasimhan 2019).

In survival analysis, continuous-time models are arguably more commonly applied than their discrete-time counterparts. However, as neural networks are parametric models, fully parametric models are typically more straightforward to integrate with the neural network frameworks than semi- and non-parametric models. This makes discrete-time survival models, which are fully parametric, interesting candidates to extend with neural networks when developing methods for time-to-event predictions.

To the best of our knowledge, Lee et al. (2018) were the first to apply modern neural networks to the discrete-time likelihood for right-censored data. Their prediction method, denoted DeepHit, parameterizes the probability mass function (PMF) of the survival distribution with a neural network and combines the log-likelihood for right-censored data with a ranking loss for improved discriminative performance. In statistical survival analysis, it is, however, more common to express the likelihood by the hazard rate. Gensheimer and Narasimhan (2019) used this form of the likelihood and parameterized the discrete-time hazard rate with a neural network. They showed that their prediction method performs well, both in terms of discrimination and calibration of the survival predictions. However, they did not compare their methodology, which they refer to as Nnet-survival, with methods that parameterize the PMF.

One aim of the present paper is to perform a systematic study of the use of neural nets in conjunction with discrete-time likelihoods for right-censored time-to-event data. In particular, we perform a systematic comparison of methods that parameterize the PMF and the discrete hazard rate.

More importantly, we show how methods that are developed for discrete-time survival data using neural networks, may be adopted as approximations for continuous-time data. In this way, we circumvent the problem that continuous-time survival models are not so easily adapted to the neural net frameworks. To this end, we have to perform a discretization of the continuous time scale; a subject that has received little attention in the literature. Immediately, it might seem reasonable to have a very fine-grained discretization scheme, allowing the approximate discretized event times to be very close to the true event times. However, the number of parameters in the neural networks typically increases with finer discretization schemes, possibly making the network overfit. Therefore, there is a balance to be considered between the number of parameters in the network and the approximation error introduced by the discretization. We consider two discretization schemes, corresponding to equidistant times or equidistant survival probabilities, and conduct a simulation study to better understand the effect of the discretization scheme and the number of time points used for the discrete-time methods.

Closely related to the discretization of a continuous time scale is the subject of interpolation. A coarse discretization grid has the benefit of reducing the number of parameters in a neural network. But the approximation error that incurs when a discrete-time method is used as an approximation for continuous-time data, becomes smaller with a finer discretization grid. By interpolating the discrete-time survival predictions, one may use a coarser discretization grid with less of an impact on the approximation error of the predictions. For this reason, two interpolation schemes are investigated in this paper. The first assumes constant density functions between the time points in the discretization grid, and the second assumes constant hazard rates between the grid points. As a modification of the latter method, we also propose a continuous-time method obtained by assuming that the continuous-time hazard rate is piecewise constant, and we compare this method with the aforementioned discrete-time methods with and without interpolation.

The paper is organized as follows. First, in Sect. 2, we consider the discrete-time likelihood for right-censored event times and discuss how the likelihood may be parameterized with neural networks. Then, in Sect. 3, continuous-time models for time-to-event data are considered, and we discuss how discretization of the continuous time scale enables the use of discrete-time survival methods for continuous-time data. Here we also present the two schemes for interpolating discrete survival functions, and we consider our continuous-time method obtained by assuming piecewise constant hazards. In Sect. 4, a simulation study is conducted to understand the impact the discretization and interpolation schemes have on the methods, and in Sect. 5, we compare the methods with existing methods for time-to-event predictions using five real-world data sets. Finally, we summarize and discuss our findings in Sect. 6. Some additional material on the simulations and the implementation of the methods are provided in the Appendix. The code for all methods, data sets, and simulations presented in this paper are available at https://github.com/havakv/pycox.

## Discrete-time models

In this section, we will restrict ourselves to models in discrete time. Then, in Sect. 3, we will discuss how discrete-time models may be used as approximations of models in continuous time.

### The discrete-time survival likelihood

Consider an individual described by its covariate vector ${\mathbf {x}}\in {\mathbb {R}}^q$. Assume that time is discrete with values $0 = \tau _0< \tau _1 < \ldots$, and let ${\mathbb {T}} = \{\tau _1, \tau _2, \dots \}$ denote the set of positive $\tau _j$’s. The time of an event is denoted $T^* \in {\mathbb {T}}$, and our goal is to model the conditional distribution of this event time given the covariate vector ${\mathbf {x}}$. The probability mass function (PMF) and the survival function for the event time are defined as

1 $$\begin{aligned} f(\tau _j \,|\,{\mathbf {x}})&= \text {P}(T^* = \tau _j \,|\,{\mathbf {x}}),\nonumber \\ S(\tau _j \,|\,{\mathbf {x}})&= \text {P}(T^*> \tau _j \,|\,{\mathbf {x}}) = \sum _{k > j} f(\tau _k \,|\,{\mathbf {x}}). \end{aligned}$$

In survival analysis, models are often expressed in terms of the hazard rate rather than the PMF. For discrete time, the hazard rate is defined as

$$\begin{aligned} h(\tau _j \,|\,{\mathbf {x}})&= \text {P}(T^* = \tau _j \,|\,T^* > \tau _{j-1}, {\mathbf {x}}) = \frac{f(\tau _j \,|\,{\mathbf {x}})}{S(\tau _{j-1} \,|\,{\mathbf {x}})} , \end{aligned}$$

and it follows that

2 $$\begin{aligned} f(\tau _j \,|\,{\mathbf {x}})&= h(\tau _j \,|\,{\mathbf {x}})\, S(\tau _{j-1} \,|\,{\mathbf {x}}), \end{aligned}$$

3 $$\begin{aligned} S(\tau _j \,|\,{\mathbf {x}})&= [1 - h(\tau _j \,|\,{\mathbf {x}})]\, S(\tau _{j-1} \,|\,{\mathbf {x}}). \end{aligned}$$

Note further that from (3) it follows that the survival function can be expressed as

4 $$\begin{aligned} S(\tau _j \,|\,{\mathbf {x}})&= \prod _{k=1}^j [1 - h(\tau _k \,|\,{\mathbf {x}})]. \end{aligned}$$

In most studies, we do not observe all event times. For some individuals, we only have a right-censored observation. To allow for censoring, we let $C^* \in {\mathbb {T}}_C = \{\tau _1, \tau _2, \ldots , \tau _m\}$ be a right-censoring time. Here $\tau _m$ defines the maximum follow-up time, at which all individuals still at risk are administratively censored. The random variables $T^*$ and $C^*$ are typically not observed directly, but instead we observe a potentially right-censored event time $T = \min \{T^*,\, C^*\}$ and an event indicator $D = \mathbbm {1}\{T^* \le C^*\}$. We here follow the common convention in survival analysis that when an event and censoring time coincide, we observe the occurrence of the event. Note that, as $C^* \le \tau _m$, we are not able to observe event times $T^*$ larger than $\tau _m$. Hence, we are restricted to model the distribution of the event times in ${\mathbb {T}}_C$.

We assume that $T^*$ and $C^*$ are conditionally independent given ${\mathbf {x}}$, and that their distributions have no parameters in common. Then we can consider, separately, the contribution to the likelihood of the event time distribution and the censoring distribution. We are, however, typically only interested in modeling the event time distribution.

Now, considering a set of *n* independent individuals, each with covariates ${\mathbf {x}}_i$, event or censoring time $t_i$, and event indicator $d_i$, the likelihood contribution of each individual *i* is given by

5 $$\begin{aligned} L_i&= {f(t_i \,|\,{\mathbf {x}}_i)}^{d_i} {S(t_i \,|\,{\mathbf {x}}_i)}^{1-d_i}. \end{aligned}$$

Using this, we can fit models by minimizing the mean negative log-likelihood

6 $$\begin{aligned} \text {loss}&= - \frac{1}{n} \sum _{i=1}^n \big \{ d_i \log [f(t_i \,|\,{\mathbf {x}}_i)] + (1-d_i) \log [S(t_i \,|\,{\mathbf {x}}_i)] \big \}. \end{aligned}$$

A useful reformulation of the loss function (6) is obtained by rewriting it in terms of the discrete hazards. To this end, let $\kappa (t) \in \{0, \ldots , m\}$ define the index of the discrete time *t*, meaning $t = \tau _{\kappa (t)}$. Using (2), (3), and (4), we can then rewrite the likelihood contribution (5) as

$$\begin{aligned} L_i = {h(t_i \,|\,{\mathbf {x}}_i)}^{d_i} \, {[1 - h(t_i \,|\,{\mathbf {x}}_i)]}^{1-d_i} \, \prod _{j=1}^{{\kappa (t_i)}-1} [1 - h(\tau _j \,|\,{\mathbf {x}}_i)]. \end{aligned}$$

With this formulation, the mean negative log-likelihood in (6) can be rewritten as

7 $$\begin{aligned} \text {loss} = - \frac{1}{n} \sum _{i=1}^n \sum _{j=1}^{\kappa (t_i)}\big \{y_{ij} \log [h(\tau _{j} \,|\,{\mathbf {x}}_i)] + (1 - y_{ij}) \log [1 - h(\tau _{j} \,|\,{\mathbf {x}}_i)] \big \}. \end{aligned}$$

Here, $y_{ij} = \mathbbm {1}\{t_i = \tau _j,\, d_i = 1\}$, so $\mathbf{y}_i = (y_{i1}, \ldots , y_{i{\kappa (t_i)}})$ is a vector of zeros with a single 1 at the event index $\kappa (t_i)$ when $t_i$ corresponds to an observed event ($d_i = 1$). We recognize this as the negative log-likelihood for Bernoulli data, or binary cross-entropy, a useful discovery first noted by Brown (1975).

With the two loss functions (6) and (7), we can now make survival models by parameterizing the PMF or the discrete hazard rate and minimizing the corresponding loss. For classical statistical models, these approaches are equivalent and have been used to obtain maximum likelihood estimates for the parameters in the PMF/hazard rate; see Tutz and Schmid (2016) for a review. We will, however, not consider classical maximum likelihood estimates, but focus on the part of the literature that fits neural networks for the purpose of time-to-event prediction, in which case the two loss functions may give different results.

### Parameterization with neural networks

A neural network $\phi ({\mathbf {x}}) \in {\mathbb {R}}^m$ is a parametric, differentiable function of a covariate vector ${\mathbf {x}}\in {\mathbb {R}}^q$ that minimizes a loss function using a gradient descent approach. While networks typically contain thousands or millions of parameters, simple models such as linear and logistic regression can also be considered neural networks. For a large number of parameters, we are usually not interested in the parameter estimates themselves, but only in the network’s predictive capabilities. While there is a vast literature on various ways to parameterize neural networks, the internal structure of the networks is not that relevant for this paper as we only consider the most standard multilayer perceptron networks, or MLP’s. So, for the purposes of this paper, we think of the network $\phi ({\mathbf {x}}) \in {\mathbb {R}}^m$ as some very flexible parametric function of the covariates ${\mathbf {x}}$. For more on MLP’s and neural networks in general see, e.g., the book by Goodfellow et al. (2016).

In the previous subsection, we saw that the survival likelihood can be expressed in terms of the PMF or the hazard rate. In the following, we will describe how to use this to create survival prediction methods by parameterizing the PMF or hazard with neural networks. In theory, as both approaches aim at minimizing the same negative log-likelihood, the methods should yield the same results. But due to the nature of neural networks, this might not be the case in practice. Contrary to most parametric statistical models, neural networks are typically overparameterized and a minimum is not obtained for the training loss. Instead, a held-out validation set is monitored, and the iterative optimization procedure is stopped when performance on this validation set starts to deteriorate. Also, considering that neural networks are well known to be sensitive to numerical instabilities, some parameterizations of a likelihood might result in better performance than others.

First, considering the hazard parametrization of the likelihood, let $\phi ({\mathbf {x}}) \in {\mathbb {R}}^m$ represent a neural network that takes the covariates ${\mathbf {x}}$ as input and gives *m* outputs. Each output $\phi _j({\mathbf {x}})$ corresponds to a discrete time-point $\tau _j$, so $\phi ({\mathbf {x}}) = {\{\phi _1({\mathbf {x}}), \ldots , \phi _m({\mathbf {x}})\}}$. As the discrete hazards are (conditional) probabilities, we apply the logistic function (sigmoid function) to the output of the network

$$\begin{aligned} h(\tau _j \,|\,{\mathbf {x}}) = \frac{1}{1 + \exp [-\phi _j({\mathbf {x}})]}, \end{aligned}$$

to ensure that $h(\tau _j \,|\,{\mathbf {x}}) \in (0, 1)$. We can estimate the hazard rate by minimizing the loss (7), and survival estimates can be obtained from (4). To the best of our knowledge, this method was first proposed by Gensheimer and Narasimhan (2019). However, if one considers the special case where $\phi _j({\mathbf {x}}) = \varvec{\beta }^T {\mathbf {x}}$, the approach is well known in the survival literature and seems to have been first addressed by Cox (1972) and Brown (1975); see also Allison (1982). The book by Tutz and Schmid (2016) gives a review of the approach.

The implementation we use in the experiments in Sects. 4 and 5 differs slightly from that of Gensheimer and Narasimhan (2019), as it was found to be numerically more stable (see Appendix B). In this paper, we will refer to the method as *Logistic-Hazard*, as coined by Brown (1975), but one can also find the term Logistic Discrete Hazard used in the statistical literature. Gensheimer and Narasimhan (2019) referred to it as *Nnet-survival*, but we will refrain from using that name as we find Logistic-Hazard to be more descriptive.

We can obtain a survival model by parameterizing the PMF in a similar manner to the Logistic-Hazard method. As for the hazards, the PMF $f(\tau _j \,|\,{\mathbf {x}})$ represents probabilities, but, contrary to the conditional probabilities that define the hazard, we now require the PMF to sum to 1. As we only observe event times in ${\mathbb {T}}_C$, we fulfill this requirement indirectly through the probability of surviving past $\tau _m$. Thus we have

8 $$\begin{aligned} \sum _{k=1}^m f(\tau _k \,|\,{\mathbf {x}}) + S(\tau _m \,|\,{\mathbf {x}}) = 1. \end{aligned}$$

Now, again with $\phi ({\mathbf {x}}) \in {\mathbb {R}}^{m}$ denoting a neural network, the PMF can be expressed as

9 $$\begin{aligned} f(\tau _j \,|\,{\mathbf {x}}) = \frac{\exp [\phi _j({\mathbf {x}})]}{1 + \sum _{k=1}^{m} \exp [\phi _k({\mathbf {x}})]}, \quad \quad \text {for } j = 1, \ldots , m. \end{aligned}$$

Note that (9) is equivalent to the softmax function (also used in multinomial logistic regression) with a fixed $\phi _{m+1}({\mathbf {x}}) = 0$. Alternatively, one could let $\phi _{m+1}({\mathbf {x}})$ vary freely, something that is quite common in machine learning, but we chose to follow the typical conventions in statistics. By combining (1) and (8), we can express the survival function as

10 $$\begin{aligned} S(\tau _j \,|\,{\mathbf {x}}) = \sum _{k=j+1}^{m} f(\tau _k \,|\,{\mathbf {x}}) + S(\tau _m \,|\,{\mathbf {x}}) \end{aligned}$$

for $$j=1,\ldots ,m-1$$, and

$$\begin{aligned} S(\tau _m \,|\,{\mathbf {x}}) = \frac{1}{1 + \sum _{k=1}^m \exp [\phi _k({\mathbf {x}})]}. \end{aligned}$$

Now, let $\sigma _j[\phi ({\mathbf {x}})]$, for $j=1,\ldots ,m+1$, denote the softmax in (9), meaning $\sigma _{m+1}[\phi ({\mathbf {x}})] = S(\tau _m \,|\,{\mathbf {x}})$. Notice the similarities to classification with $m+1$ classes, as we are essentially classifying whether the event is happening at either time $\tau _1, \ldots , \tau _m$ or later than $\tau _m$. However, due to censoring, the likelihood is *not* the cross-entropy. Instead, by inserting (9) and (10) into (6), we get the mean negative log-likelihood

11 $$\begin{aligned} \text {loss}&= -\frac{1}{n} \sum _{i=1}^n \left( d_i \log [\sigma _{\kappa (t_i)}(\phi ({\mathbf {x}}_i)) ] + (1-d_i) \log \left[ \sum _{k={\kappa (t_i)}+1}^{m+1} \sigma _k(\phi ({\mathbf {x}}_i)) \right] \right) , \end{aligned}$$

where ${\kappa (t_i)}$ still denotes the index of individual *i*’s event or censoring time, that is, $t_i = \tau _{\kappa (t_i)}$. This is essentially the same negative log-likelihood as presented by Lee et al. (2018). Note, however, that contrary to the work by Lee et al. (2018) the negative log-likelihood in (11) allows for survival past time $\tau _m$. Some numerical improvements of the implementation are addressed in Appendix B. We will refer to this method simply by *PMF* as this term is unambiguously discrete, contrary to the term *hazard* which is used both for discrete and continuous time.

As a side note, the Multi-task logistic regression (Yu et al. 2011), and the neural network extension of this method (Fotso 2018), can be shown to be a PMF model by considering a cumulative sum of the linear predictor, or in the neural network case, the cumulative sum of the output of the network. Details are given in Appendix C.

## Continuous-time models

In the following, we no longer consider the time scale to be discrete, but instead consider continuous-time models, where $T^*, C^* > 0$, and we let $T = \min \{T^*, C^*\}$ and $D = \mathbbm {1}\{T^*\le C^*\}$ be as before. Let $\tau$ denote the maximum possible value of $C^*$, so that $P(C^* \le \tau ) = 1$. Hence, a potentially right-censored observation *T* is in the interval $\mathopen {(} 0,\, \tau \mathclose {]}$. Instead of a PMF, we now have the density function $f(t \,|\,{\mathbf {x}})$ and the continuous-time survival function

$$\begin{aligned} S(t \,|\,{\mathbf {x}}) = \text {P}(T^* > t \,|\,{\mathbf {x}}) = \int _t^\tau f(z \,|\,{\mathbf {x}})\, dz + S(\tau \,|\,{\mathbf {x}}). \end{aligned}$$

Furthermore, the continuous-time hazard rate is a non-negative function of the time (no longer restricted to [0, 1]),

12 $$\begin{aligned} h(t \,|\,{\mathbf {x}}) = \frac{f(t \,|\,{\mathbf {x}})}{S(t \,|\,{\mathbf {x}})} = \lim _{\Delta t \rightarrow 0} \frac{\text {P}(t \le T^* < t + \Delta t \,|\,T^* \ge t, {\mathbf {x}})}{\Delta t}. \end{aligned}$$

As a result, we can express the survival function in terms of the cumulative hazard $H(t \,|\,{\mathbf {x}}) = \int _{0}^t h(z \,|\,{\mathbf {x}})\, dz$,

13 $$\begin{aligned} S(t \,|\,{\mathbf {x}}) = \exp [-H(t \,|\,{\mathbf {x}})]. \end{aligned}$$

This yields the continuous-time version of the likelihood contribution in (5),

14 $$\begin{aligned} L_i = {f(t_i \,|\,{\mathbf {x}}_i)}^{d_i}\, {S(t_i \,|\,{\mathbf {x}}_i)}^{1-d_i} = {h(t_i \,|\,{\mathbf {x}}_i)}^{d_i}\, \exp [-H(t_i \,|\,{\mathbf {x}}_i)]. \end{aligned}$$

In what follows, we will first discuss how we can apply the discrete-time methods from Sect. 2.2 for continuous-time data. We will here address how time can be discretized to fit the discrete-time model formulation, and how to interpolate an estimated discrete survival function for continuous-time predictions. Then, we will propose a new continuous-time method by assuming that the hazard in (12) is piecewise constant.

### Discretization of the time scale

**Fig. 1:**
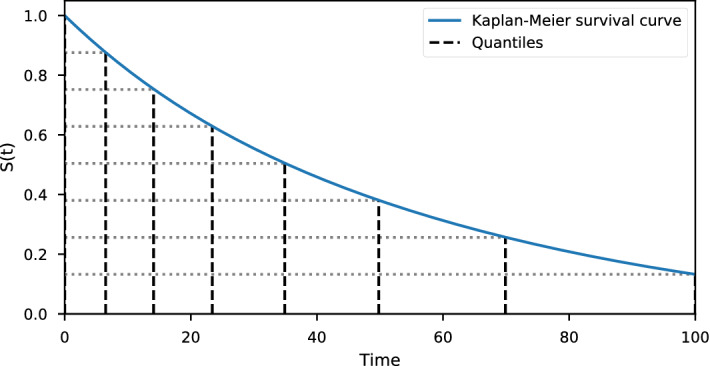
Illustration of the Kaplan–Meier based discretization scheme. The quantiles of the Kaplan–Meier curve are used as the grid points

Both the PMF and Logistic-Hazard methods require time to be discrete on the form $0 = \tau _0< \tau _1< \cdots < \tau _m$. Hence, to apply the methods to continuous-time data, we need to perform some form of discretization of the time scale. Possibly the most obvious way to discretize time is to make an equidistant grid in $[0, \tau ]$ with *m* grid points. An alternative, that we explore in this paper, is to make a grid based on the distribution of the event times. By disregarding covariates and estimating the marginal survival function *S*(*t*) with the Kaplan–Meier estimator, we obtain a general trend of event times. With ${{\hat{S}}}(t)$ denoting the Kaplan–Meier survival estimates, we can make a grid from the quantiles of the estimates, $1 = {{\hat{S}}}(0) = \zeta _0> \zeta _1> \cdots > \zeta _m = {{\hat{S}}}(\tau )$. We will assume that each interval has the same decrease in the survival estimate, so that $\zeta _j - \zeta _{j+1} = (1 - {{\hat{S}}}(\tau )) / m$. The corresponding time grid, $\tau _1< \cdots < \tau _m$, is obtained by letting $\tau _j$ be the smallest value of *t* such that ${{\hat{S}}}(t) \le \zeta _j$. We will then obtain a more dense grid in intervals with more events, and a less dense grid in intervals with fewer events. This is illustrated in Fig. 1, where we can see that the grid becomes coarser as the slope of the survival curve becomes less steep.

The discrete-time methods assume that all events and censorings occur at the $\tau _j$’s, so, when performing the discretization, we move all event times in an interval to the end of that interval while censored times are moved to the end of the previous interval. This means that for $\tau _{j-1} < T_i \le \tau _j$, we replace $T_i$ by $\tau _j$ if $D_i = 1$, and by $\tau _{j-1}$ if $D_i = 0$. Our reason for this choice is that this is typically how the times are recorded. Consider a study where we are only able to make observations at times $\tau _1< \tau _2< \cdots < \tau _m$. For a censored observation, $\tau _{j-1}$ is the last point in time where the individual was recorded alive, while for an observed event, $\tau _j$ is the first point in time for which the individual was recorded with the event.

### Interpolation for continuous-time predictions

**Fig. 2:**
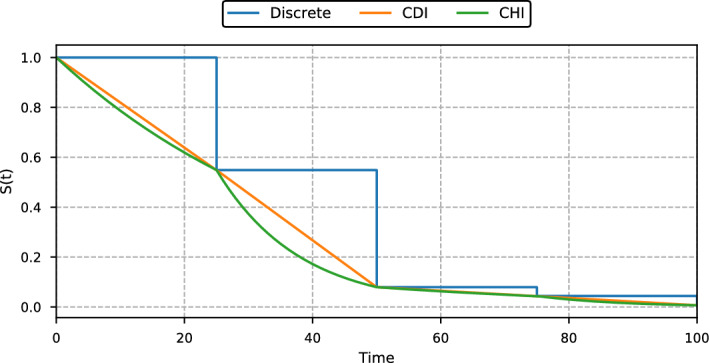
Survival estimates by a discrete model for 5 grid points. The three lines represent the discrete survival estimates and the two interpolation schemes in Sect. 3.2: The constant density interpolation (CDI) and constant hazard interpolation (CHI)

When discrete-time survival methods are applied to continuous-time data, the survival estimates become a step function with steps at the grid points; see the blue curve in Fig. 2. Consequently, for coarser grids, it might be beneficial to interpolate the discrete survival estimates. To this end, we propose two simple interpolation schemes that fulfill the monotonicity requirement of the survival function. The first assumes that the probability density function is constant in each time interval $\mathopen {(} \tau _{j-1},\, \tau _{j} \mathclose {]}$, while the second scheme assumes constant hazard in each time interval. We will refer to the schemes as *constant density interpolation* (CDI) and *constant hazard interpolation* (CHI). Note that the two interpolation schemes correspond to piecewise linear and piecewise exponential survival estimates, as illustrated in Fig. 2.

### A piecewise constant continuous-time hazard parametrization

We now propose a continuous-time method by parameterizing the hazards in (14). As for the constant hazard interpolation, we will let the continuous-time hazard be piecewise constant. Disregarding neural networks, this model was first proposed by Holford (1976), and further developed by Friedman (1982) who found that piecewise constant hazards yield a likelihood proportional to that of a Poisson likelihood.

Consider a partition of the time scale $0 = \tau _0< \tau _1< \cdots < \tau _m = \tau$, and let $\kappa (t)$ denote the interval index of time *t* such that $t \in \mathopen {(} \tau _{{\kappa (t)}-1},\, \tau _{\kappa (t)} \mathclose {]}$ (this is slightly different from the discrete case where we had $t = \tau _{\kappa (t)}$). If we assume that the hazard is constant within each interval, we can express the hazard as the step function $h(t \,|\,{\mathbf {x}}) = \eta _{\kappa (t)}({\mathbf {x}})$ for a set of non-negative functions $\{\eta _1({\mathbf {x}}), \ldots , \eta _m({\mathbf {x}})\}$. For $\Delta \tau _j = \tau _j - \tau _{j-1}$, we can now express the cumulative conditional hazard as

$$\begin{aligned} H(t \,|\,{\mathbf {x}}) = \left( \sum _{j=1}^{{\kappa (t)}- 1} \eta _j({\mathbf {x}})\, \Delta \tau _j\right) + \eta _{\kappa (t)}({\mathbf {x}})\, (t - \tau _{{\kappa (t)}-1 }). \end{aligned}$$

Inserting this into (14) yields the likelihood contribution for individual *i*,

$$\begin{aligned} L_i = {\eta _{\kappa (t_i)}({\mathbf {x}}_i)}^{d_i}\, \exp \left[ -\eta _{\kappa (t_i)}({\mathbf {x}}_i)\, (t - \tau _{{\kappa (t_i)}- 1})\right] \prod _{j=1}^{{\kappa (t_i)}- 1} \exp \left[ -\eta _j({\mathbf {x}}_i)\, \Delta \tau _{j}\right] . \end{aligned}$$

What remains is to parameterize the hazard with a neural network. However, to avoid passing all the $\tau _j$’s to the loss function, we let the network instead parameterize the functions $\tilde{\eta }_j({\mathbf {x}}) = \eta _j({\mathbf {x}})\, \Delta \tau _j$. This allows us to rewrite the likelihood contribution as

$$\begin{aligned} L_i&= {\left( \frac{{{{\tilde{\eta }}}_{\kappa (t_i)}({\mathbf {x}}_i)}}{\Delta \tau _{\kappa (t_i)}}\right) }^{d_i}\, \exp \left[ -{{\tilde{\eta }}}_{\kappa (t_i)}({\mathbf {x}}_i)\, \rho (t_i) \right] \prod _{j=1}^{{\kappa (t_i)}- 1} \exp \left[ -{{\tilde{\eta }}}_j({\mathbf {x}}_i)\right] \\&\propto {{{\tilde{\eta }}}_{\kappa (t_i)}({\mathbf {x}}_i)}^{d_i}\, \exp \left[ -{{\tilde{\eta }}}_{\kappa (t_i)}({\mathbf {x}}_i)\, \rho (t_i) \right] \prod _{j=1}^{{\kappa (t_i)}- 1} \exp \left[ -{{\tilde{\eta }}}_j({\mathbf {x}}_i)\right] , \end{aligned}$$

where

15 $$\begin{aligned} \rho (t) = \frac{t - \tau _{{\kappa (t)}-1}}{\Delta \tau _{\kappa (t)}}, \end{aligned}$$

is the proportion of interval ${\kappa (t)}$ at time *t*.

As before, let $\phi ({\mathbf {x}}) \in {\mathbb {R}}^m$ denote a neural network. To ensure that ${\tilde{\eta }}_j({\mathbf {x}})$ is non-negative, we could have used ${\tilde{\eta }}_j({\mathbf {x}}) = \exp [\phi _j({\mathbf {x}})]$. However, for better numerical stability we prefer to use the softplus function

16 $$\begin{aligned} {\tilde{\eta }}_j({\mathbf {x}}) = \log (1 + \exp [\phi _j({\mathbf {x}})]). \end{aligned}$$

Now, again considering *n* independent individuals, each with covariates ${\mathbf {x}}_i$, observed event or censoring time $t_i$, and event indicator $d_i$, our model can be fitted by minimizing the mean negative log-likelihood

$$\begin{aligned} \text {loss}&= - \frac{1}{n}\sum _{i=1}^n \left( d_i\, \log {\tilde{\eta }}_{\kappa (t_i)}({\mathbf {x}}_i) - {\tilde{\eta }}_{\kappa (t_i)}({\mathbf {x}}_i)\, \rho (t_i) - \sum _{j=1}^{{\kappa (t_i)}- 1} {\tilde{\eta }}_j ({\mathbf {x}}_i) \right) , \end{aligned}$$

and estimates for the survival function can be obtained by

17 $$\begin{aligned} S(t \,|\,{\mathbf {x}}) = \exp [- H(t \,|\,{\mathbf {x}})] = \exp [-{\tilde{\eta }}_{\kappa (t)}({\mathbf {x}})\, \rho (t)] \prod _{j=1}^{{\kappa (t)}-1} \exp [-{\tilde{\eta }}_j({\mathbf {x}})], \end{aligned}$$

where $\rho (t)$ is given by (15). We will refer to this method as the *piecewise constant hazard* method, or *PC-Hazard*. Even though this is a continuous-time method, we still need to decide the set of $\tau _j$’s that define the intervals. Therefore, the discretization techniques discussed in Sect. 3.1 are also relevant for this method.

## Simulations

To get a better understanding of the methodologies discussed in Sects. 2 and 3, we perform a simulation study where we vary the size of the training sets, the discretization scheme, and the number of grid points used for discretization. Gensheimer and Narasimhan (2019) performed a similar study to evaluate the effect of discretization on their Logistic-Hazard method with the conclusion that there were no differences in performance. However, their simulations were quite simple (only one binary covariate), their only performance metric was the Harrell Jr et al. (1982) concordance at 1-year survival, and they did not include any interpolation of the survival estimates. For this reason, we find that further investigations are warranted.

We generate simulated survival times by sequentially sampling from discrete-time hazards defined on a fine grid of time points. The hazards are specified through their logit transforms, as this enables us to use functions in ${\mathbb {R}}$ while still obtaining hazards in (0, 1). The logit hazards, $g(t \,|\,{\mathbf {x}}) = \text {logit}[h(t \,|\,{\mathbf {x}})]$, are defined as

18 $$\begin{aligned} g(t \,|\,{\mathbf {x}})&= \alpha _1({\mathbf {x}})\, g_\text {sin}(t \,|\,{\mathbf {x}}) + \alpha _2({\mathbf {x}})\, g_\text {con}(t \,|\,{\mathbf {x}}) + \alpha _3({\mathbf {x}})\, g_\text {acc}(t \,|\,{\mathbf {x}}), \end{aligned}$$

where

$$\begin{aligned} g_\text {sin}(t \,|\,{\mathbf {x}})&= \gamma _1({\mathbf {x}}) \sin \big (\gamma _2({\mathbf {x}}) [ t + \gamma _3({\mathbf {x}})]\big ) + \gamma _4({\mathbf {x}}),\\ g_\text {con}(t \,|\,{\mathbf {x}})&= \gamma _5({\mathbf {x}}), \\ g_\text {acc}(t \,|\,{\mathbf {x}})&= \gamma _6({\mathbf {x}}) \cdot t - 10, \end{aligned}$$

and

$$\begin{aligned} \alpha _i({\mathbf {x}})&= \frac{\exp (\gamma _{i+6}({\mathbf {x}}))}{\sum _{j=1}^3 \exp (\gamma _{j+6}({\mathbf {x}}))}, \quad \text {for } i=1, 2, 3. \end{aligned}$$

**Fig. 3:**
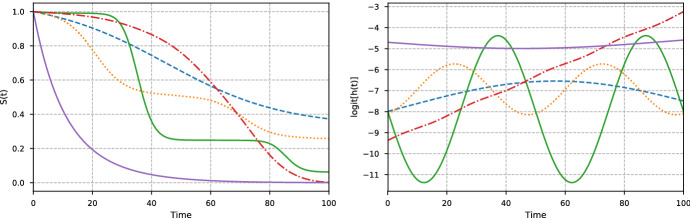
Examples from the simulation study in Sect. 4. The left figure shows examples of 5 simulated survival curves, while the right figure shows the corresponding logit hazards. The examples are selected to illustrate the richness of event time distributions that are expressed by the covariates in the simulated data

Each of the three functions $g_\text {sin}(t \,|\,{\mathbf {x}})$, $g_\text {con}(t \,|\,{\mathbf {x}})$, and $g_\text {acc}(t \,|\,{\mathbf {x}})$ are constructed to give a specific contribution to the hazards: $g_\text {con}(t \,|\,{\mathbf {x}})$ gives a constant hazard for a set of covariates, $g_\text {acc}(t \,|\,{\mathbf {x}})$ allows for a hazard that increases with time, and $g_\text {sin}(t \,|\,{\mathbf {x}})$ enables periodic patterns in the hazards. With this combination, we are able to represent a variety of event time distributions. Each function $\gamma _k({\mathbf {x}})$ uses five of the covariates in ${\mathbf {x}}$, totaling a covariate vector ${\mathbf {x}}$ of size 45. The functions $\gamma _k({\mathbf {x}})$ are defined in Appendix A. We let the discrete time scale consist of 1,000 equidistant points between 0 and 100 so that $\tau _0=0$, $\tau _1 = 0.1$, $\tau _2 = 0.2$, ..., $\tau _{1000} = 100$. Knowing the hazards, the true survival function can be obtained with (4). In Fig. 3 we show five examples of logit hazard rates and their corresponding survival functions. Note that even though we simulate our data using a discrete-time model, the time-grid is so fine that this mimics simulation from a continuous-time model. The full details of this simulation study are given in Appendix A.

### Simulation setup, hyperparameter tuning, and evaluation

We created three training sets of size 3000, 10,000, and 50,000, a validation set of size 10,000 (for hyperparameter tuning), and a test set of size 100,000. For the training and validation sets, we included a censoring distribution with constant hazard resulting in 37% censoring. The full uncensored test set is used for evaluation. For the discretization of the time scale, we applied both the equidistant scheme and the Kaplan–Meier quantiles, each with 5, 25, 100, and 250 grid points.

The three prediction methods under investigation, described in Sects. 2.2 and 3.3, all consist of the same “base” neural network $\phi ({\mathbf {x}}) \in {\mathbb {R}}^m$, but have individual output layers and functions transforming $\phi ({\mathbf {x}})$ to survival estimates. These final transforms do, however, not include any parameters or hyperparameters. For a fair comparison, we therefore perform the same hyperparameter search of $\phi ({\mathbf {x}})$ for all models. As an extensive search is unfeasible for such models, we only consider MLP networks with ReLU activation functions, batch normalization, and dropout between each layer. The size of the networks is controlled by the number of layers and the number of nodes in each layer, which we assume to be the same for all layers. Our choices are motivated by an attempt to represent the most standard MLP’s. For more on MLP’s and neural networks in general see, e.g., the book by Goodfellow et al. (2016).

We performed a hyperparameter grid search over 1, 2, 4, and 8 hidden layers; 16, 64, and 256 nodes; and dropout of 0 (no dropout) and 0.5. Each net was trained with a batch size of 256 and the AdamWR optimizer (Loshchilov and Hutter 2019) with cycle length 1, where, at each restart, the cycle length was doubled and the learning rate was multiplied by 0.8. Learning rates were found using the methods proposed by Smith (2017). The methods’ respective loss functions (negative log-likelihoods), computed on the validation set, were used for selecting the best set of hyperparameters. The hyperparameter tuning was repeated 10 times, giving 10 fitted models for each combination of method, grid size, discretization scheme, and training set size. In general, the discretization scheme, both granularity and discretization method, are hyperparameters. In the simulation experiments, however, we are interested in comparing the performance across discretization schemes, and to this end we obtain a model for each scheme. But when we in Sect. 5 compare performance on real data, we need to include the discretization scheme in the hyperparameter tuning.

For evaluating the predictive performance of the methods, we consider two metrics on the uncensored held-out test set. The first metric is the average mean squared error (MSE) between the survival estimates and the true survival function at all 1000 time points $\tau _1, \ldots , \tau _{1000}$

19 $$\begin{aligned} \text {MSE} = \frac{1}{100,000} \sum _{i=1}^{100,000} \frac{1}{1000}\sum _{j=1}^{1,000} {\left( {{\hat{S}}}(\tau _j \,|\,{\mathbf {x}}_i) - S(\tau _j \,|\,{\mathbf {x}}_i) \right) }^2. \end{aligned}$$

Here ${{\hat{S}}}(\tau _j \,|\,{\mathbf {x}}_i)$ and $S(\tau _j \,|\,{\mathbf {x}}_i)$ are the estimated and true survival functions, respectively, for individual *i* (in the test set) at time $\tau _j$. So, in this regard, the discrete-time survival estimates are represented by step functions, as illustrated in Fig. 2. Note that the MSE (19) is only applicable for simulated data, as the true survival functions $S(\tau _j \,|\,{\mathbf {x}}_i)$ are not known in real-world applications.

The second metric is the time-dependent concordance (Antolini et al. 2005), which evaluates a method’s ability to correctly rank individuals’ survival estimates according to their event times. This is achieved by estimating the probability of correctly ranking two arbitrary individuals

$$\begin{aligned} \text {P}({\hat{S}}(T_i \,|\,{\mathbf {x}}_i)< {\hat{S}}(T_i \,|\,{\mathbf {x}}_j) \,|\,T_i < T_j, D_i = 1), \end{aligned}$$

where $T_i$ and $T_j$ are the known potentially right-censored event times of individuals *i* and *j*. In other words, if individual *i* experiences the event of interest while individual *j* is still at risk, a good set of predictions should give a lower survival estimate for individual *i* than individual *j* at the event time of individual *i*. Note that, contrary to the MSE, larger values for the concordance are considered better.

### Comparison of discrete-time methods

We start by comparing the two discrete methods from Sect. 2.2, that parameterize the PMF and the discrete-time hazards. We refer to them as PMF and Logistic-Hazard, respectively.

**Fig. 4:**
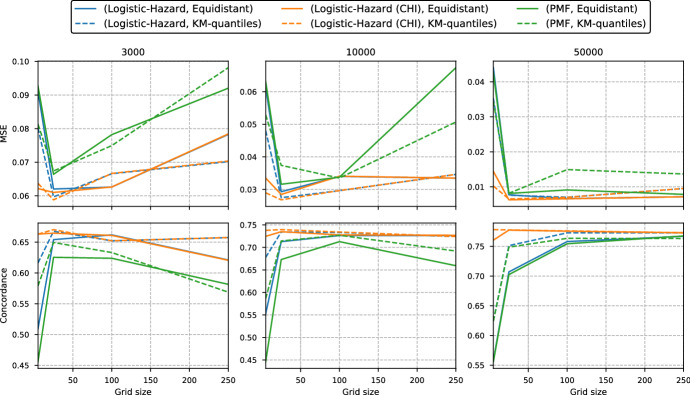
Median MSE and concordance for each grid size in the simulation study in Sect. 4.2. The number above each plot gives the size of the training set. The full lines use an equidistant grid, while the dotted lines use Kaplan–Meier quantiles for discretization. Note that the plots are not on the same scale

In Fig. 4 we plot the median test scores of the two methods versus the grid size used for discretization. The number above each plot gives the size of the training set used to fit the methods. The full lines represent equidistant grids, while the dotted lines are from grids obtained with quantiles from Kaplan–Meier survival estimates. We have also included the constant hazard interpolation (CHI) of the survival estimates from the Logistic-Hazard method (see Sect. 3.2).

For the smallest training set of size 3000 we see that the best performance (smallest MSE and highest concordance) is obtained with a grid of size 25 and that the finer grids of size 100 and 250 result in much worse performance (higher MSE and lower concordance). For the two larger training sets, the finer grids have generally performance on par with or better than that of grid of size 25. This is reasonable as coarser grids require fewer parameters in the neural networks, and the networks with very fine grids are therefore more likely to overfit the data. Nevertheless, the coarsest grid of size 5 seems to only work well for the interpolated estimates, and does very poorly for the discrete estimates. The discretization grids from Kaplan–Meier quantiles seem to give slightly better scores than the equidistant grids for the Logistic-Hazard with the coarsest grids; in particular for the smaller training sets. This difference is, however, quite small. Comparing the discrete survival estimates from Logistic-Hazard (blue lines) with the CHI estimates (orange lines), we see that the two lines overlap for finer grids. This is expected as the effect of interpolation decreases as the grids become finer.

In general, the PMF method does not perform as well as the Logistic-Hazard.

### Comparison of interpolation schemes

**Fig. 5:**
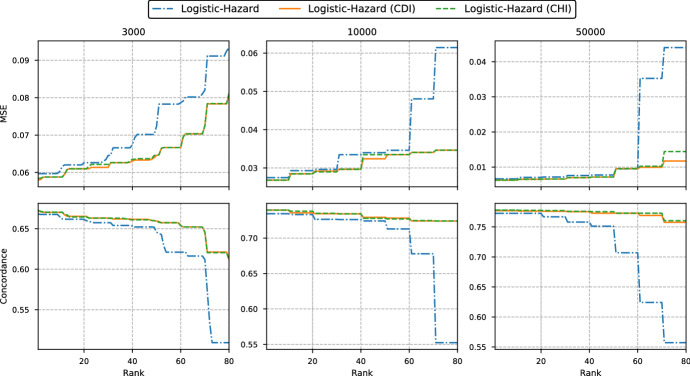
MSE and concordance from the simulation study in Sect. 4.3. The scores are plotted from best to worst. The number above each plot gives the size of the training set. Note that the plots are not on the same scale

In the following, we compare the interpolation schemes for the discrete-time hazard method Logistic-Hazard. The experiments are not shown for the PMF method as the results are very similar.

In Sect. 3.2 we presented two methods for interpolation of discrete survival estimates. The first assumes constant density in each interval (denoted CDI for constant density interpolation), while the second assumes constant hazard in each interval (denoted CHI for constant hazard interpolation). In our simulation study, we have four grid sizes and two discretization schemes. As the hyperparameter tuning was repeated 10 times this gives 80 fitted models for each method on each data set. In Fig. 5, we plot the scores of these 80 models sorted from best to worst, as this both tells us the best performance, in addition to the stability of the methods. The figure contains results from the discrete survival estimates (Logistic-Hazard), the constant density interpolation (CDI), and the constant hazard interpolation (CHI).

Clearly, there is almost no difference in performance between the two interpolation schemes, while the discrete estimates have slightly worse best-case performance and much worse worst-case performance. So the interpolation primarily helps with stability in performance across discretization schemes, but similar performance can be obtained for discrete predictions given careful hyperparameter tuning.

As the two interpolation schemes perform the same, we will in the further simulations only include the CHI estimates as they and the continuous-time PC-Hazard method both assume constant hazard rate, simplifying the comparison between the methods.

### Comparison with PC-Hazard

**Fig. 6:**
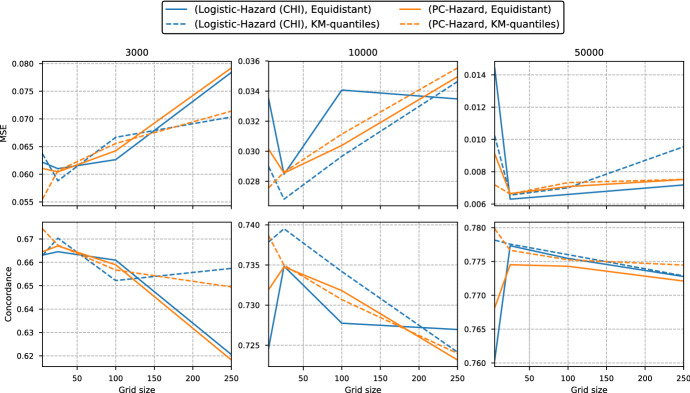
Median MSE and concordance for each grid size of the simulation study in Sect. 4.4. The number above each plot gives the size of the training set. The full lines use an equidistant grid, while the dotted lines use Kaplan–Meier quantiles for discretization. Note that the plots are not on the same scale

Finally, we compare the previous methods with our proposed continuous-time hazard method from Sect. 3.3, PC-Hazard. In Fig. 6 we plot the MSE and concordance for the interpolated Logistic-Hazard (CHI) method and the continuous-time PC-Hazard method. Note that the y-axis is compressed compared to the previous figures. First, we notice that PC-Hazard does better for the coarsest grid with only five grid points, while Logistic-Hazard (CHI) typically performs best with 25 grid points. In general, the differences in performance between the two models are very small. We note, however, that the best performing model for the smallest training set is the PC-Hazard with a Kaplan–Meier grid of size 5, both in terms of MSE and concordance. Finally, we again see that the Kaplan–Meier quantiles seem to give slightly better performance than the equidistant discretization when the grids are coarse.

**Fig. 7:**
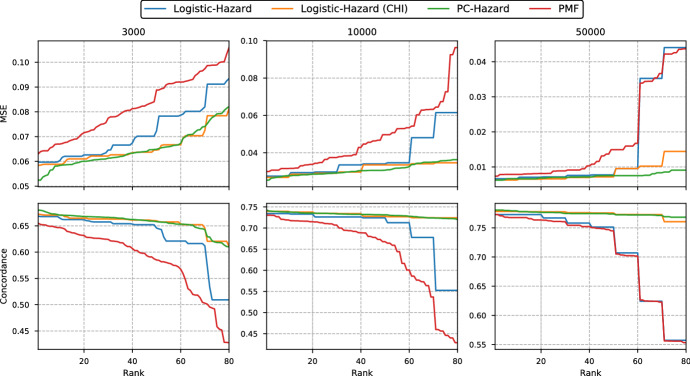
MSE and concordance from the simulation study in Sect. 4. The scores are plotted from best to worst. The number above each plot gives the size of the training set. Note that the plots are not on the same scale

In Fig. 7 in Appendix A, we have included a plot of the same type as Fig. 5 for the Logistic-Hazard (CHI) method, the Logistic-Hazard method, the PMF method, and the PC-Hazard method. The figure again shows that the PMF method performs slightly worse than the other methods, while the PC-Hazard method performs similarly to the Logistic-Hazard (CHI) estimates.

### Summary of simulations

To summarize the results of the simulations, we have shown that the size of the discretization grid (number of $\tau _j$’s) has a large impact on the performance of the methods, and therefore needs to be carefully tuned. Finer grids enable the methods to reduce bias in the predictions but require more parameters in the neural networks (higher variance). By defining the discretization grid with Kaplan–Meier quantiles, the performance for the coarser grids typically improves, while it has no apparent effect for finer grids.

Interpolation of the discrete-time survival estimates alleviates some of the sensitivity to the discretization scheme. For the coarser grids, interpolation was generally found to improve performance, while it does affect the performance for finer grids. The performance of the two proposed interpolation schemes, CHI and CDI, was more or less indistinguishable.

Comparing the three methods, we found that PMF did not perform as well as the Logistic-Hazard, both in terms of best-case performance and stability to discretization-grid configurations. PC-Hazard was found to be competitive with the interpolated Logistic-Hazard method and even performed better for the smallest training set. But the differences between all methods were small, and the size of the training sets and the grid size were shown to have a much larger impact on the performance than the choice of method.

## Experiments with real data

We now compare the methods discussed in this paper to other methods in the literature, in particular DeepHit (Lee et al. 2018), DeepSurv (Katzman et al. 2018), Cox-Time (Kvamme et al. 2019), CoxCC (Kvamme et al. 2019), Random Survival Forests (RSF, Ishwaran et al. 2008), and a regular Cox regression.

We conduct the comparison on five common real-world data sets: the Study to Understand Prognoses Preferences Outcomes and Risks of Treatment (SUPPORT), the Molecular Taxonomy of Breast Cancer International Consortium (METABRIC), the Rotterdam tumor bank and German Breast Cancer Study Group (Rot. & GBSG), the Assay Of Serum Free Light Chain (FLCHAIN), and the National Wilm’s Tumor Study (NWTCO). Katzman et al. (2018) made the first three data sets available in their python package DeepSurv, and we have made no further preprocessing of the data. FLCHAIN and NWTCO were made available in the survival package of R (Therneau 2015), but we use the same version of FLCHAIN as Kvamme et al. (2019). No alterations were made to the NWTCO data set. The size, the number of covariates, and the proportion of censored individuals in each data set are given in Table 1.

**Table 1:** Data sets for comparing survival methods

Data set	Size	Covariates	Prop. censored
FLCHAIN	6524	8	0.70
METABRIC	1904	9	0.42
NWTCO	4028	6	0.86
Rot. & GBSG	2232	7	0.43
SUPPORT	8873	14	0.32

### Hyperparameter tuning and evaluation

The experiments were conducted by five-fold cross-validation. For each split, a separate hyperparameter search was conducted, meaning that for each method we may end up with separate hyperparameters for each split.

For hyperparameter tuning, we set aside 20% of the data in each split as a validation set (corresponding to 16% of the full data set). For each method considered, we used the tuning criteria proposed in the original papers for choosing the best hyperparameters.

For the methods presented in this paper, we cannot use the validation loss for the hyperparameter search, as done in the simulations in Sect. 4.1. This is because we now need to include the discretization scheme as part of the hyperparameter search, and the losses (negative log-likelihoods) are dependent on the granularity of the discretization scheme (dependent on the number of output nodes). Instead, we will use the integrated Brier score (IBS) by Graf et al. (1999) computed over 100 equidistant points between the minimum and maximum observed times in the validation set. The IBS considers both discrimination and calibration of the survival estimates, and accounts for censored individuals by weighting the score by the inverse of the estimated censoring distribution.

For evaluation, we will consider the IBS in addition to the time-dependent concordance (Antolini et al. 2005). In contrast to the IBS, the concordance only evaluate the discriminative capabilities of a method’s predictions. It is interesting to study both metrics as there might be a trade-off between well-calibrated estimates and good discriminative performance. For these real data sets, we do not know the true survival function and we can therefore not use the MSE (19) reported in the simulation studies.

The experiments were conducted using the same hyperparameter search and training strategy as presented in Section 6.1 of the paper by Kvamme et al. (2019), but we decrease the learning rate by 0.8 at the start of each cycle, as this was found to give more stable training. For the methods requiring discretization of the time scale, the hyperparameter search considered grid sizes of 5, 25, 50, 100, and 200, both with equidistant spacing and by using the Kaplan–Meier quantiles. The best parameter configuration for each method in each of the five cross-validation splits was fitted 10 times, and we calculated the median concordance and integrated Brier score (IBS) of the 10 repetitions and averaged these over the five folds.

### Results

The results are presented in Tables 2 and 3. In terms of concordance, we see that DeepHit and PC-Hazard perform very well. The three Logistic-Hazard methods and Cox-Time all perform close to PC-Hazard, while the PMF, RSF and the other Cox methods perform slightly worse. The concordances of the two proposed interpolation schemes, CHI and CDI, are very similar, but the CDI method tends to give slightly better scores. There does, however, not seem to be much performance gain in interpolation for the concordance.

**Table 2:** Concordance from 5-fold cross-validation on real-world data sets

Model	FLCHAIN	METABRIC	NWTCO	Rot. & GBSG	SUPPORT
Cox Regression	0.790	0.626	0.706	0.664	0.599
CoxCC	0.792	0.647	0.711	0.670	0.614
DeepSurv	0.792	0.640	0.709	0.674	0.615
Cox-Time	**0**.**793**	0.664	0.709	0.674	0.630
RSF	0.784	0.651	0.705	0.668	0.632
DeepHit	0.791	**0**.**675**	0.710	0.675	**0**.**639**
PMF	0.786	0.632	0.710	0.669	0.627
Logistic-Hazard	0.792	0.658	0.704	0.670	0.625
Logistic-Hazard (CHI)	0.790	0.656	0.714	0.673	0.628
Logistic-Hazard (CDI)	0.790	0.660	0.700	0.676	0.630
PC-Hazard	0.791	0.655	**0**.**716**	**0**.**679**	0.628

Largest values for each data are set in bold

**Table 3:** Integrated Brier score from 5-fold cross-validation on real-world data sets

Model	FLCHAIN	METABRIC	NWTCO	Rot. & GBSG	SUPPORT
Cox Regression	0.0961	0.183	0.0791	0.180	0.218
CoxCC	0.0924	0.173	0.0745	0.171	0.213
DeepSurv	0.0919	0.175	0.0745	0.170	0.213
Cox-Time	0.0925	0.173	0.0753	0.170	**0**.**212**
RSF	0.0928	0.175	0.0749	0.170	0.213
DeepHit	0.0929	0.186	0.0758	0.184	0.227
PMF	0.0924	0.174	0.0748	**0**.**169**	0.213
Logistic-Hazard	0.0918	**0**.**172**	0.0742	0.171	0.213
Logistic-Hazard (CHI)	0.0919	0.173	**0**.**0738**	0.170	0.213
Logistic-Hazard (CDI)	**0**.**0917**	**0**.**172**	0.0741	0.170	**0**.**212**
PC-Hazard	0.0918	**0**.**172**	**0**.**0738**	**0**.**169**	**0**.**212**

Smallest values for each data are set in bold

Examining the IBS in Table 3 (smaller is better) we again find that PC-Hazard performs very well. But now, DeepHit does quite poorly. This is expected as DeepHit is designed for discrimination rather than well-calibrated estimates (see Kvamme et al. 2019). In general, the PMF, the RSF, and the three proportional Cox methods seem to have slightly higher IBS than the Hazard methods, but again the differences are quite small. Cox-Time performs quite well on all data sets except for FLCHAIN and NWTCO. Comparing the interpolation schemes of Logistic-Hazard, it seems that CDI still performs slightly better than CHI, although both are quite close to the discrete estimates of Logistic-Hazard.

In summary, all three methods discussed in this paper are competitive with existing survival methodology. However, the interpolated Logistic-Hazard and the PC-Hazard seem to give the most stable high performance considering both discrimination and calibration.

## Discussion

In this paper, we have explored survival methodology built on neural networks for discrete-time data, and how it can be applied for continuous-time prediction. We have compared two existing discrete-time survival methods that minimize the negative log-likelihood of right-censored event times, where the first method (Lee et al. 2018) parameterize the event time probability mass function (PMF), while the second method (Gensheimer and Narasimhan 2019) parameterize the discrete hazard rate (Logistic-Hazard). Through empirical studies of simulated and real data sets, we found that the Logistic-Hazard method typically performs better than the PMF parametrization, both in terms of discrimination and calibration of the survival predictions.

We proposed two interpolation schemes for the discrete methods. Both schemes were found to improve predictions for methods with a coarse discretization of the time scale. In particular, as coarser discretization reduces the number of network parameters, the interpolation schemes gave the largest improvements when applied to smaller data sets. The two interpolation schemes were found to perform very similarly.

We also proposed a new continuous-time method that assumes constant hazard in predefined time intervals (PC-Hazard). The method was found to perform very well compared to existing methods, both in terms of discrimination and calibration. Furthermore, in a simulation study, we found that the method continued to perform better for coarser discretization grids than the interpolated Logistic-Hazard method. This was particularly beneficial for the smallest training set in the simulation study.

All three methods investigated in this paper need some form of discretization or coarsening of the time scale. In that regard, we proposed a simple scheme that uses the quantiles of the event time distribution estimated by Kaplan–Meier, and showed through simulations that the quantile-based grids typically outperformed equidistant grids for coarser grids.

In summary, we found that all three methods perform quite similarly, and the choice of discretization has a larger impact on performance than the choice of method. For the discrete methods, interpolation of the survival predictions will typically make the performance less sensitive to the discretization scheme.

Some interesting further development of our work would be to extend the models to allow for left-truncated event times and competing risks. While these are common topics in survival analysis, the literature on neural network extensions is quite limited.

Finally, we have only considered time-to-event prediction given the information that is available at the outset of a study. One could also be interested in predicting the remaining time until an event given observation of time-dependent covariates and that the event has not yet happened by a time $t_0$. The landmarking approach of van Houwelingen and Putter (2011) offers one possible framework for such dynamic predictions.

## Appendix A More on the simulations

In the following, we include additional information about the simulation study in Sect. 4. We start by explaining in detail how the data sets were created, and in Sect. A.2 we give some additional results.

### Discrete-time survival Simulations from logit hazards

The simulated survival data sets were generated by drawing from the discrete hazard $h(t \,|\,{\mathbf {x}})$ across times $t \in \{0.1, 0.2, \ldots , 100\}$. The discrete hazard was defined through the logit hazard $g(t \,|\,{\mathbf {x}}) \in {\mathbb {R}}$,

$$\begin{aligned} h(t \,|\,{\mathbf {x}}) = \frac{1}{1 + \exp [- g(t \,|\,{\mathbf {x}})]}, \end{aligned}$$

ensuring that $h(t \,|\,{\mathbf {x}}) \in (0,\, 1)$. In (18), we defined the logit hazard $g(t \,|\,{\mathbf {x}})$ in terms of a set of functions $\gamma _i({\mathbf {x}})$, for $i = 1, \ldots 9$. In order to describe how the $\gamma _i({\mathbf {x}})$’s are defined, let ${\mathbf {x}}_{(j)}$ define a subset of the covariates ${\mathbf {x}}$ and let ${{\tilde{x}}}_j$ be a linear combination of this subset, ${{\tilde{x}}}_j = {\mathbf {x}}_{(j)}^T \varvec{\beta }_j$ for $j=1, \dots , 9$, where the subsets are non-overlapping and of equal size (if the subsets are of size *m*, we have ${\mathbf {x}}\in {\mathbb {R}}^{9 m}$). The $\gamma _i({\mathbf {x}})$’s are defined as

$$\begin{aligned} \gamma _1({\mathbf {x}})&= 5 {{\tilde{x}}}_1, \\ \gamma _2({\mathbf {x}})&= \frac{2\,\pi }{100} \cdot 2^{\lfloor \frac{5}{2}({{\tilde{x}}}_2 + 1) - 1 \rfloor },\\ \gamma _3({\mathbf {x}})&= 15 {{\tilde{x}}}_3, \\ \gamma _4({\mathbf {x}})&= 2 {{\tilde{x}}}_4 - 6 - |\gamma _1({\mathbf {x}})|, \\ \gamma _5({\mathbf {x}})&= \frac{5}{2} ({{\tilde{x}}}_5 + 1) - 8, \\ \gamma _6({\mathbf {x}})&= \frac{1}{1 + \exp [- \frac{6}{2} ({{\tilde{x}}}_6 + 1) + 5]}, \\ \gamma _7({\mathbf {x}})&= 5 ({{\tilde{x}}}_7 + 0.6),\\ \gamma _8({\mathbf {x}})&= 5 {{\tilde{x}}}_8,\\ \gamma _{9}({\mathbf {x}})&= 5 {{\tilde{x}}}_{9}, \end{aligned}$$

where $\lfloor z \rfloor$ is the floor operation. We draw $\tilde{x}_j \overset{iid}{\sim } \text {Unif}[-1, \, 1]$, and $\beta _k \overset{iid}{\sim } N(0,\, 1)$. The forms of the $\gamma _i({\mathbf {x}})$’s have been chosen to obtain reasonable survival functions. In particular, $\gamma _2({\mathbf {x}})$ ensures that the number of periods is a multiple of 2, as we wanted to introduce a more restricted seasonality pattern in hazard than any arbitrary period.

Finally, denoting the covariates in ${\mathbf {x}}_{(j)} \in {\mathbb {R}}^m$ as $x_{j,1}, x_{j, 2}, \ldots x_{j, m}$, we draw $x_{j,k}$ while ensuring ${\mathbf {x}}_{(j)}^T \varvec{\beta }_j = {{\tilde{x}}}_j$ through the following scheme: For known $\varvec{\beta }_j \in {\mathbb {R}}^m$, we draw $x_{j,k}$ conditionally such that

$$\begin{aligned} \left( {{\tilde{x}}}_j - \sum _{i=1}^{k} x_{j, i}\, \beta _{j, i} \right) \,|\,{{\tilde{x}}}_j, x_{j,1}, \ldots , x_{j, k-1} \sim \text {Unif}[-1,\, 1], \quad \text {for } k=1,\ldots ,m-1. \end{aligned}$$

Hence, we sample $u_{j,k} \overset{iid}{\sim } \text {Unif}[-1,\, 1]$ for $k=1, \ldots , m-1$, and set $u_{j,k} = {{\tilde{x}}}_j - \sum _{i=1}^{k} x_{j, i}\, \beta _{j, i}$, giving the covariates

$$\begin{aligned} x_{j,k} = {\left\{ \begin{array}{ll} \frac{1}{\beta _{j,1}} \left( {{\tilde{x}}}_j - u_{j, 1}\right) , &{} \text {if } k = 1\\ \frac{1}{\beta _{j, k}}\left( u_{j, k-1} - u_{j, k}\right) , &{} \text {if } k=2, \ldots , m-1\\ \frac{1}{\beta _{j, m}} \left( {{\tilde{x}}}_j - \sum _{i=1}^{m-1} x_{j, i} \beta _{j, i} \right) , &{} \text {if } k = m. \end{array}\right. } \end{aligned}$$

Using this scheme, it is straightforward to change the number of covariates without affecting the hazards. The code for generating these simulations is available at https://github.com/havakv/pycox.

### Additional simulation results

We here present some additional results from the simulation study in Sect. 4.3. Recall that each method is fitted 80 times (4 grids $\times$ 2 discretization schemes $\times$ 10 repetitions). In the same manner as in Fig. 5, we plot in Fig. 7 the MSE and concordance for the Logistic-Hazard, Logistic-Hazard (CHI), PC-Hazard, and PMF, where the scores of the 80 models are sorted from best to worst.

We again see that PC-Hazard and the Logistic-Hazard (CHI) perform better than the discrete estimates of Logistic-Hazard and PMF. Furthermore, Logistic-Hazard seems to generally perform better than the PMF method. We still find that for the best grid configurations, the differences between all models are very small. But we reiterate that, for practical purposes, it is quite desirable to have stable performance for a variety of hyperparameter configurations.

## Appendix B Implementation details

The implementations of the survival methods described in Sects. 2 and 3 are slightly different from the mathematical notation. This is because we also need to consider numerical stability. An implementation of the methods can be found at https://github.com/havakv/pycox.

For the PMF parameterization, we used the log-sum-exp trick

$$\begin{aligned} \log \left( \sum _j \exp (z_j) \right) = \gamma + \log \left( \sum _j \exp (z_j-\gamma ) \right) , \end{aligned}$$

where $\gamma = \max _j(z_j)$, to ensure that we only take the exponential of non-positive numbers. Hence, by rewriting the loss (11) in terms of $\phi _j({\mathbf {x}})$, with $\phi _{m+1}({\mathbf {x}}) = 0$ and $\gamma _i = \max _j (\phi _j({\mathbf {x}}_i))$, we obtain

$$\begin{aligned} \text {loss} =&- \frac{1}{n} \sum _{i=1}^n d_i [\phi _{\kappa (t_i)}({\mathbf {x}}_i) - \gamma _i] + \frac{1}{n}\sum _{i=1}^n \log \left( \sum _{j=1}^{m+1} \exp [\phi _j({\mathbf {x}}_i) - \gamma _i]\right) \\&- \frac{1}{n} \sum _{i=1}^n (1-d_i) \log \left( \sum _{j= {\kappa (t_i)}+1}^{m+1} \exp [\phi _j({\mathbf {x}}_i)-\gamma _i] \right) . \end{aligned}$$

For the discrete hazard parametrization, we note that the loss in (7) is written as a sum over binary cross-entropy terms. This allows us to rely on existing implementations of binary cross-entropy to ensure numerical stability. In practice, these implementations use the log-sum-exp trick on the $\phi _j({\mathbf {x}})$ (the logits).

Finally, for the continuous hazard parametrization, we use existing implementations of the softplus function which uses a linear function over a certain threshold, meaning $\log (1 + \exp [z]) \approx z$ for large values of *z*. However, we also note that for $z \approx 0$, we have that $\log (1 + z) \approx z$. Hence, for $\phi _{\kappa (t_i)}({\mathbf {x}}_i) \ll 0$ we use that

$$\begin{aligned} \log {\tilde{\eta }}_{\kappa (t_i)}({\mathbf {x}}_i) = \log [\log (1 + \exp [\phi _{\kappa (t_i)}({\mathbf {x}}_i)])] \approx \phi _{\kappa (t_i)}({\mathbf {x}}_i). \end{aligned}$$

## Appendix C Multi-task logistic regression as PMF

Multi-task logistic regression, proposed by Yu et al. (2011), provides a generalization of the binomial log-likelihood to jointly model the sequence of binary labels $Y_j = \mathbbm {1}\{T^*\le \tau _j\}$. This means that $Y = (y_1, \ldots , y_m)$ is a sequence with zeros for every time $\tau _j$ up to the event time, followed by one’s. Then

C.1 $$\begin{aligned} \text {P}(Y = (y_1, \dots , y_m) \,|\,{\mathbf {x}}) = \frac{\exp \left[ \sum _{k=1}^m y_k \psi _k({\mathbf {x}})\right] }{1 + \sum _{k=1}^{m} \exp \left[ \sum _{l=k}^m \psi _l({\mathbf {x}})\right] }. \end{aligned}$$

Yu et al. (2011) only consider linear predictors $\psi _k({\mathbf {x}}) = {\mathbf {x}}^T \varvec{\beta }_k$, but this was extended to a neural network by Fotso (2018). The parameters of $\psi _k({\mathbf {x}})$ are found by minimizing the negative log-likelihood in (6).

As $f(\tau _j \,|\,{\mathbf {x}}) = \text {P}(Y=(y_1, \ldots , y_m) \,|\,{\mathbf {x}})$, where $y_k = \mathbbm {1}\{k \ge j\}$, the expression in (C.1) can be written as

$$\begin{aligned} f(\tau _j \,|\,{\mathbf {x}})&= \frac{\exp \left[ \sum _{k=j}^m \psi _k({\mathbf {x}})\right] }{1 + \sum _{k=1}^{m} \exp \left[ \sum _{l=k}^m \psi _l({\mathbf {x}})\right] } = \frac{\exp [\phi _j({\mathbf {x}})]}{1 + \sum _{k=1}^{m} \exp [\phi _k({\mathbf {x}})]}, \end{aligned}$$

where $\phi _j({\mathbf {x}}) = \sum _{k=j}^m \psi _k({\mathbf {x}})$. Hence, the multi-task logistic regression is equivalent to the PMF in (9), but where $\phi _j({\mathbf {x}})$ is the (reverse) cumulative sum of the output of the network $\psi ({\mathbf {x}}) \in {\mathbb {R}}^m$. To the extent of our knowledge, there are no benefits to this extra cumulative sum. Instead, it simply requires unnecessary computations, and, for large *m*, it can cause numerical instabilities.
